# Mutations in *dock1* disrupt early Schwann cell development

**DOI:** 10.1186/s13064-018-0114-9

**Published:** 2018-08-08

**Authors:** Rebecca L. Cunningham, Amy L. Herbert, Breanne L. Harty, Sarah D. Ackerman, Kelly R. Monk

**Affiliations:** 1 Department of Developmental Biology, Washington University School of Medicine, St. Louis, MO 63110 USA; 2 Vollum Institute, Oregon Health and Science University, Portland, OR 97239 USA; 3 Institute of Neuroscience, University of Oregon, Eugene, OR 97403 USA

**Keywords:** *dock1*, Schwann cell development, Myelination, Zebrafish

## Abstract

**Background:**

In the peripheral nervous system (PNS), specialized glial cells called Schwann cells produce myelin, a lipid-rich insulating sheath that surrounds axons and promotes rapid action potential propagation. During development, Schwann cells must undergo extensive cytoskeletal rearrangements in order to become mature, myelinating Schwann cells. The intracellular mechanisms that drive Schwann cell development, myelination, and accompanying cell shape changes are poorly understood.

**Methods:**

Through a forward genetic screen in zebrafish, we identified a mutation in the atypical guanine nucleotide exchange factor, *dock1*, that results in decreased myelination of peripheral axons. Rescue experiments and complementation tests with newly engineered alleles confirmed that mutations in *dock1* cause defects in myelination of the PNS. Whole mount *in situ* hybridization, transmission electron microscopy, and live imaging were used to fully define mutant phenotypes.

**Results:**

We show that Schwann cells in *dock1* mutants can appropriately migrate and are not decreased in number, but exhibit delayed radial sorting and decreased myelination during early stages of development.

**Conclusions:**

Together, our results demonstrate that mutations in *dock1* result in defects in Schwann cell development and myelination. Specifically, loss of *dock1* delays radial sorting and myelination of peripheral axons in zebrafish.

**Electronic supplementary material:**

The online version of this article (10.1186/s13064-018-0114-9) contains supplementary material, which is available to authorized users.

## Background

Myelin, a lipid-rich multi-membrane structure, is an innovation of jawed vertebrates that enables the efficient conduction of action potentials. Schwann cells are the myelinating glia of the peripheral nervous system (PNS), and one Schwann cell myelinates one axonal segment. Schwann cells are derived from the neural crest and undergo a distinct series of developmental stages [1, 2]. These developmental stages of Schwann cells require migration as well as unique and substantial changes in cell shape. Schwann cell precursors (SCPs) migrate great distances longitudinally down peripheral nerves. SCPs develop into immature Schwann cells, which undergo a unique process called radial sorting in which Schwann cells extend processes into axon bundles and select an axon to myelinate [3]. Prior to myelination, Schwann cells wrap themselves 1–1.5 times around a selected axon segment in what is termed the pro-myelinating state. A mature Schwann cell extends and wraps its membrane to form a myelin sheath around an axonal segment. Cytoskeletal dynamics are needed to facilitate these different stages of Schwann cell development and extensive changes in cell shape, but the intracellular intermediates between extracellular signals and the remodeling of the Schwann cell cytoskeleton are not well defined.

The Rho-GTPase Rac1 is well known for its role in facilitating cell shape changes through regulating polymerization of the actin cytoskeleton and mediates Schwann cell development [4]. In Schwann cells, differential levels of Rac1 direct when a Schwann cell stops migrating and begins radial sorting and myelination [5]. Schwann cell-specific ablation of Rac1 in a mouse model causes delays in radial sorting and myelination, as well as aberrant Schwann cell process extension [5–7]. Furthermore, Rac1 can function downstream of β1-integrin in Schwann cells performing radial sorting [5]; however, the intracellular mechanisms that influence the temporal and spatial activation of Rac1 following extracellular signaling during Schwann cell development are not well understood.

Guanine nucleotide exchange factors (GEFs) have the ability to temporally and spatially regulate the activation of RhoGTPases, such as Rac1, because many GEFs can regulate the same RhoGTPase [8]. Roles of specific GEFs during distinct stages of Schwann cell development are beginning to be understood and help to broaden our knowledge of how extracellular signals are translated to intracellular signals in order to facilitate alterations in Schwann cell shape and movement [9–12]. In addition to canonical GEFs, atypical GEFs also have the ability to activate RhoGTPases. One such family of atypical GEFs, the Dock1-related GEFs, is composed of 11 family members, including Dock1 (also known as Dock180). Dock1 is highly evolutionarily conserved across species and can specifically bind and activate Rac1 [13–15]. In vitro and in vivo studies in various model organisms have shown that Dock1 influences a variety of cytoskeletal-related cell processes such as phagocytosis and cell migration [16–19]. Thus, Dock1 represents an ideal intracellular candidate to study for a role in cell shape regulation.

Although Dock1 has been studied in several biological contexts and is expressed in Schwann cells [20], a role for Dock1 has not yet been described in Schwann cell myelination. The ability of Dock1 to initiate changes in cell shape to facilitate phagocytosis and cell migration makes Dock1 an attractive candidate to investigate for a role in regulated cell shape changes throughout the development of Schwann cells, particularly during stages of radial sorting and myelination, when Rac1 levels most influence Schwann cell biology [4]. Two other members of the Dock1 family, Dock7, which activates the RhoGTPase Cdc42 [20], and Dock8 [21], which can activate Cdc42 and Rac1, have been shown to influence SCP migration through in vitro and in vivo knockdown experiments. Therefore, other members of the Dock1 family may also be key intracellular signals regulating the timing of Schwann cell development.

In this study, we utilized zebrafish to study Schwann cell myelination [22], and we identify and characterize Dock1 as a regulator of early Schwann cell myelination. Although previous morpholino experiments in zebrafish have implicated *dock1* in myoblast development and vasculature morphogenesis [23–25], a role for Dock1 in Schwann cell development has not been examined. In a screen for genetic regulators of myelination, we identified an early stop codon in *dock1* that causes decreased expression of a mature myelin marker, *myelin basic protein* (*mbp*), in the PNS. Transmission electron microscopy (TEM) revealed that fewer axons are myelinated in mutants during early stages of myelin development, while axon number is not affected. We determined that SCP cell number and migration is not affected in *dock1* mutants. Instead, radial sorting is delayed and early markers of myelination are reduced. These data suggest that Dock1 may contribute to the timely process extension of Schwann cells required for radial sorting and myelination.

## Methods and materials

### Zebrafish lines and rearing conditions

Zebrafish were reared in accordance with the Washington University IRB and animal protocols and were raised in the Washington University Zebrafish Consortium (http://zebrafish.wustl.edu/husbandry.htm). Zebrafish were crossed as either pairs or harems, and embryos were subsequently raised at 28.5 °C in egg water (5 mM NaCl, 0.17 mM KCl, 0.33 mM CaCl_2_, 0.33 mM MgSO_4_). Larvae were staged at hours post fertilization (hpf) and days post fertilization (dpf). The following mutant and transgenic strains were utilized in this study: *dock1*^*stl145*^, *dock1*^*stl365*^, *dock1*^*stl366*^, *Tg(sox10(4.9):nls-eos)* [26], *Tg(foxd3:gfp)* [27], and *Tg(kdlr:mcherry*) [28]. Homozygous *dock*^*stl145*^ fish are viable as adults, therefore maternal zygotic (MZ) *dock1*^*stl145*^ animals were generated by crossing a *dock*^*stl145/stl145*^ female with a *dock1*^*stl145/+*^ male.

### Genotyping

To identify adult and larval zebrafish for either rearing or phenotypic analyses, the following primers were used to amplify a region of interest by PCR: *stl145* F: 5’-CATAGGCGTTCTTCACTGAG-3′ and R: 5’-CGTATTTCCCACTAAACAGC-3′*, stl365* F: 5’-GCAGCCACTTTAAAGCTTCCCG-3′ and R: 5’-GCTGCTTACCTTGCCCTTGTC-3′*,* and *stl366* F: 5’-CCAGTGCCTCACTTCATATCTCC-3′ and R: 5’ CTCTTAGTCTCACGCAACACTCATG-3′*.* After PCR, a restriction enzyme digest assay was performed and the resulting fragments were analyzed on a 3% agarose gel. The *stl145* C-to-T mutation disrupts a BstNI site so that the wild-type PCR product is cleaved into 48 and 527 base pair (bp) products, and the mutant PCR product is 575 bp. The *stl365* allele contains a one bp insertion that disrupts an EcoRV binding site so that the wild-type PCR product is cleaved into 86 and 159 bp products, and the mutant PCR product is 245 bp. The *stl366* allele contains a 13-bp deletion that disrupts a HpyCH4III site so that the wild-type PCR product is cleaved into 323 bp and 165 bp products, and the mutant PCR product is 488 bp.

### Zebrafish mutant strain generation

*dock1*^*stl145*^ was identified in a forward genetic screen described previously in [29, 30]. Phenotypically wild-type and mutant 5 dpf larvae were pooled and extracted DNA was sent for whole genome sequencing at the Genome Technology Access Center (GTAC) at Washington University. The wild-type to mutant allele ratio was determined using a bioinformatics pipeline generated in-house, and a SNP subtraction analysis suggested that *dock1* was most likely the gene of interest [30]. *dock1* was confirmed as the gene responsible for the *stl145* mutant phenotype through rescue experiments and complementation tests using two other *dock1* mutant alleles, *dock1*^*stl365*^ and *dock1*^*stl366*^, which were generated by TALENs. The TALEN targeter tool (https://tale-nt.cac.cornell.edu/) and GoldyTALEN kit [31] were utilized to build each TALEN in a pCS2+ backbone. The repeat variable domains chosen for each *stl365* TALEN arm and *stl366* TALEN arm were: *stl365* left arm: NN HD NG HD NI HD HD NG NN NI HD NN HD NI NN NI NN NI NN NI; *stl365* right arm: HD NG NG NG NN NI NN NG NG NN NI HD HD HD NG NN NI NN NG; *stl366* left arm: NN NG NG NI NG NI NG NG HD NI NG HD NG NN NI NI NN NN NI NN; *stl366* right arm: NN HD NG NG NI NI NI HD NI NG NI HD NG NN NI HD HD HD NN HD. The TALEN constructs were transcribed with the mMESSAGE mMACHINE SP6 ULTRA Kit (Ambion) and equal concentrations (~ 50 pg) of left and right arm mRNA were injected into 1-cell stage wild-type embryos. Lesions that were successfully transmitted to the F0 germline were identified by restriction enzyme digest analysis as described above. Mutant bands were gel extracted using a QIAquick Gel Extraction kit (Qiagen) and then Sanger sequenced to identify the lesion.

### Posterior lateral line nerve (PLLn) dissection and RNA isolation

Posterior lateral line nerves (PLLn) were dissected from 6-month-old adult zebrafish. Animals were euthanized in ice water until gill motion ceased for 5 min, followed by transection of the hindbrain. Using angled forceps, the skin was pulled back from behind the operculum on both sides of the animal to expose the PLLn. Small spring-loaded dissection scissors were used to cut the PLLn near the operculum and then forceps were used to gently remove the nerve by slowly pulling the nerve toward the anterior of the fish. Both nerves were transferred to microcentrifuge tubes sitting on dry ice and then flash frozen in liquid nitrogen and stored at − 80 °C. To isolate RNA, 40 nerves were pooled from 20 different 6-month-old adult zebrafish and total RNA was obtained using standard TRIzol (Life Technologies) RNA extraction, with the exception of the homogenization method. Nerves were pooled into a total of 500 μl TRIzol and then thoroughly homogenized following these steps in succession: vortexing for 30 s, disruption with a plastic-tipped electric homogenizer for 1 min, and passaged through a syringe and successively smaller needles (22.5 and 27 gauge), 10 times each.

### RT-PCR

To make cDNA, 1 μg of total RNA was reverse transcribed using the High Capacity cDNA Reverse Transcription Kit (Applied Biosystems) using random hexamers as per manufacturer instructions. RT-PCR for *dock1* was performed on adult PLLn cDNA. The following primers were used: F: 5’-CGGAGTGGCCGTCTACAACTATG-3′ (bordering exons 1 and 2) and R: 5’-CAAGCCGGAAACACACCCTTC-3′ (bordering exons 3 and 4). Milli-Q water was used as a substrate for a control RT-PCR.

### Rescue experiment

Full-length zebrafish *dock1* was cloned into pCS2+ using Gibson Assembly. *dock1* was amplified in two pieces from zebrafish cDNA using a Phusion mastermix (NEB) and the following primers: part 1: F: 5’-TCTTTTTGCAGGATCCCATAGAGAAGCGAGAAAAAGTGTG-3′ and R: 5’-CTCCATGATGATCTGCACGTG-3′ and part 2: F: 5’-TCAGCGACATACTGGAGGTGC-3′ and R: 5’-TAATACGACTCACTATAGTTGAGGTGTCAGCTGCTTTTCCG-3′. Gibson Assembly was then performed using an in-house Gibson reaction mixture (gifted by the Solnica-Krezel lab, Washington University in St. Louis). Briefly, the fragments were gel extracted and purified using the QIAquick Gel Purification Kit (Qiagen). 30 ng of pCS2+, linearized with Clal and Xbal, were combined with 5-fold excess of the *dock1* PCR fragments and 15 μl of the Gibson Assembly enzyme-reagent mixture. The mixture was incubated at 50 °C for 1 h and then 10 μl were transformed into DH5 alpha cells and plated on ampicillin plates. Subsequent colonies were grown, miniprepped with a Qiagen Kit, and Sanger sequenced. Synthetic mRNA for injection was generated by linearizing *dock1* in pCS2+ with Not1 and then transcribing with the mMESSAGE mMACHINE SP6 ULTRA Kit (Ambion). Approximately 120 pg of *dock1* mRNA in 2 nl was injected into 1-cell stage embryos generated from a *dock1*^*stl145*^ heterozygous in-cross. In situ hybridization for *mbp* was performed and scoring of expression was performed blinded to genotype.

### Whole mount in situ hybridization and qualitative scoring

Whole mount in situ hybridization (WISH) was performed as described [32, 33] on larvae treated with 0.003% phenylthiourea from 24 h post fertilization (hpf) to inhibit pigmentation until fixation in 4% paraformaldehyde. The previously characterized riboprobes used in this study were: *sox10* [34], *krox20* [35], and *mbp* [36]. All phenotypes were scored with the scorer blinded to genotype. The PLLn was scored for strength of staining: “strong” = strong and consistent expression along the entirety of the PLLn; “reduced” = consistent but reduced *mpb* expression along PLLn; and “strongly reduced” = patches of *mbp* expression or no expression, similar to scoring as performed previously [37]. “Strong” *mbp* expression was assigned a value of 3, “reduced,” a value of 2, and “strongly reduced,” a value of 1 to code each phenotype as a number for a Chi-squared anaylsis.

### Transmission Electron microscopy and quantifications

TEM was performed on 3 dpf, 5 dpf, and 21 dpf cross-sections of the PLLn according to standard protocols [38, 39]. Larvae were cut between body segments 5 and 6 and juvenile 21 dpf fish were cut immediately posterior to the heart. A Jeol JEM-1400 (Jeol USA) electron microscope and AMT V601 digital camera were used to image samples. Quantification of percent myelinated axons, sorted axons, total axon number, and number of Schwann cell nuclei was performed on the entire cross section of the PLLn. The scorer was blinded to genotype, and quantification was performed manually as described previously [37].

### Lifeact microinjections and live imaging

One-cell stage zebrafish embryos were injected with ~ 15–20 ng of *sox10:Lifeact-RFP* (a gift from the Lyons lab, University of Edinburgh) and 25 ng of transposase mRNA. 1 dpf larvae were then screened for expression of *sox10:Lifeact-RFP* in Schwann cells at 24 hpf. For live-imaging, larvae were anesthetized in Tricaine and embedded in 0.8% agarose on a 35 mm glass bottom dish filled with 0.2% Tricaine and covered with a 22 × 22 mm^2^ coverslip on top of vacuum grease [40]. The larvae were then imaged with a Zeiss LSM 880 confocal microscope at 20× for 3 h at 3 min intervals. Still images were captured with a Zeiss LSM 880 II Airyscan FAST confocal microscope at 40xW with a 1.8 zoom. To examine blood vesssls, 4 dpf larvae with *Tg(kdlr:mcherry)* were imaged at 13.5× with a Nikon SMZ18 fluorescent dissecting microscope.

### Eos Photoconversion and quantification of Schwann cell number

*Tg(foxd3:gfp);dock1*^*stl145/+*^ fish were crossed to *Tg(sox10(4.9):nls-eos);dock1*^*stl145/+*^ fish and offspring were screened for both transgenes at 1 dpf. At 2 dpf, larvae were placed in 0.8% low-melt agarose and mounted for imaging as described above. Before counting, larvae were individually exposed to 30 s of UV light using the DAPI filter with the 20× objective of a Zeiss LSM 880 confocal microscope. The number of GFP and RFP positive cells along the PLLn spanning ~ 8 body segments were the counted manually in ImageJ. The observer was blinded to genotype.

### Neuromast labeling and quantification

3 dpf larvae derived from a *dock1*^*stl145*^ heterozygous in-cross were incubated with 50 μl of DASPEI (40 mg/ 100 mL in distilled water) in 4 mL of egg water for 15 min at room temperature. The DASPEI solution was removed and replaced with fresh egg water. The number of neuromasts along the PLLn were counted under a fluorescent dissecting microscope using a GFP filter.

### Immunohistochemistry

Immunohistochemistry for acetylated tubulin was performed as described in [32] with mouse anti-acetylated alpha-tubulin used at a dilution of 1:1000 (Sigma). Larvae were fixed at 4 dpf and were derived from a *dock1*^*stl145*^ heterozygous in-cross. Heavy myosin within somites was detected with chicken MF 20 antibody at a dilution of 1:20 (Developmental Studies Hybridoma Bank). MF 20 was deposited to the DSHB by Fischman, D.A. (DSHB Hybridoma Product MF 20). For MF 20 staining, embryos were fixed at 1 dpf in 4% paraformaldehyde for 1 h and washed twice with 1X PBS for 10 min. Samples were then blocked with 0.05% Triton in PBS and 10% goat serum and then incubated with MF 20 in block overnight at 4 °C. After incubation, larvae were washed twice with PBS and then incubated secondary antibody in PBS for 2 h at room temperature. Primary antibodies were detected IgG2b with secondary antibody conjugated to either Alexa 568 or 488 (Invitrogen) at a 1:2000 dilution. Immunostained larvae were imaged with a Nikon SMZ18 fluorescent dissecting microscope.

### Statistical analyses

GraphPad Prism 7 was utilized to perform statistical tests. Unpaired t-tests with Welch’s correction were used to test significance of all TEM, neuromast number, and Schwann cell number data. A Chi-squared analysis was utilized to determine significance for all WISH data. Phenotypes of “strong,” “reduced,” and “strongly reduced” were assigned a number of 3, 2, or 1, respectively, in order to compare phenotypes with a Chi-squared analysis. An unpaired t-test with Welch’s correction showed no significant difference between wild-type and heterozygous animals; therefore, for TEM, WISH, neuromast, and Schwann cell number data, wild-type and heterozygous animals were combined as controls.

## Results

### Mutations in *dock1* result in decreased *myelin basic protein* expression in the peripheral nervous system

**Fig. 1:**
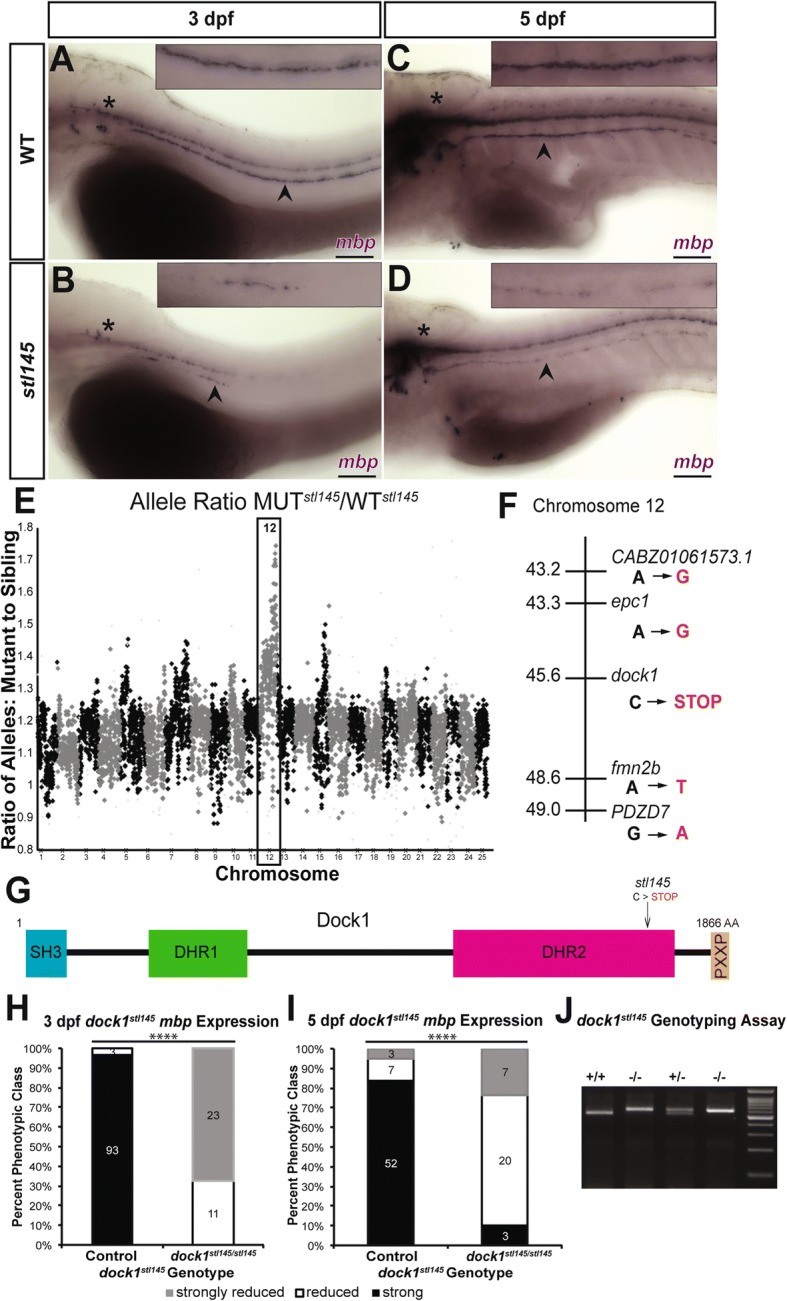
*stl145* mutants exhibit decreased *mbp* expression in the PNS. **a-d)** Lateral views of *mbp* expression by WISH. Arrowheads indicate the PLLn. Asterisks indicate the central nervous system (CNS). Inset panels show a magnified view of the PLLn. Scale bars = 100 μm. **a)** *mbp* at 3 dpf is strongly expressed in the PLLn of control larva (*n* = 93/96). **b)** *stl145* mutants at 3dpf exhibit reduced *mbp* expression in the PLLn (*n* = 34). **c)** *mbp* expression is strongly expressed in the PLLn of control larva at 5 dpf (*n* = 52/62). **d)** *stl145* mutants at 5 dpf express *mbp*, but at reduced levels compared to control siblings (*n* = 27/30). **e)** Analysis of whole genome sequencing data showed that chromosome 12 exhibited the highest mutant to wild-type allele ratio. **f)** Within the most highly linked region of chromosome 12, *dock1* was the only gene that contained an early stop codon. **g)** A schematic of the protein structure of Dock1 and the location of the *stl145* lesion. The SH3 and proline rich domains can bind adaptor proteins. The DHR-1 domain interacts with PtdIns(3,4,5)P_3_ and the DHR-2 domain is the catalytic domain can that catalyzes the exchange of GDP for GTP in Rac1. **h-i)** Quantification of WISH for *mbp* at 3 dpf **(h)** and 5 dpf **(i)**, respectively, based on phenotypic classes and genotypes for the *stl145* lesion. **** *p* < 0.0001, Chi-squared analysis. **j)** Genotyping assay for the *stl145* lesion. The PCR amplified product is digested with BstN1 and run on a 3% agarose gel

**Fig. 2:**
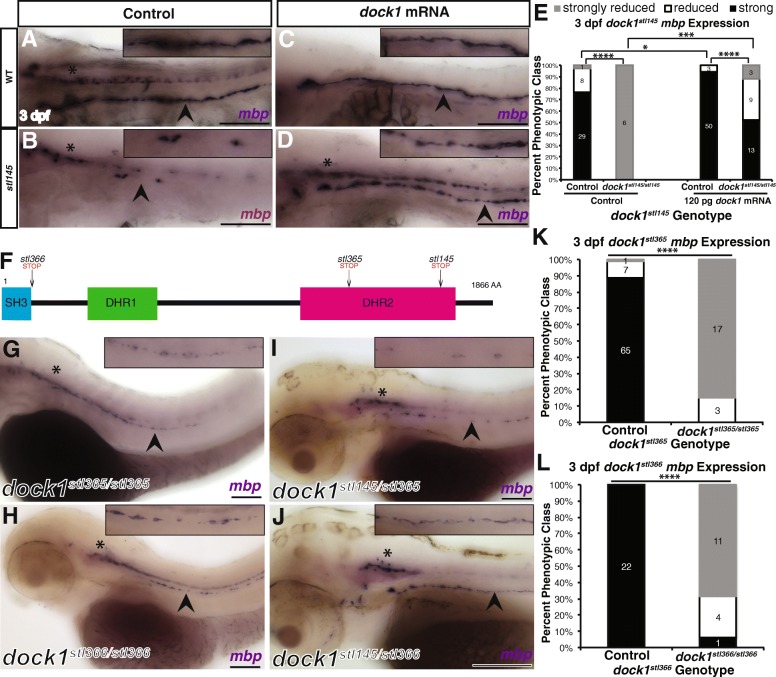
Mutations in *dock1* cause decreased *mbp* expression in the PNS. **a-d)** Lateral views of *mbp* expression by WISH at 3 dpf. Arrowheads indicate PLLn. Asterisks indicates the CNS. Inset panels show a magnified view of PLLn. Scale bars = 100 μm**. a)** Control larvae robustly express *mbp* in the PLLn (*n* = 29/38). **b)** *dock1*^*stl145*^ homozygous mutants exhibit strongly reduced *mbp* expression in the PLLn (*n* = 6/6). **c)** Control larvae injected with *dock1* mRNA exhibit strong expression of *mbp* in the PLLn (*n* = 50/53). **d)** *dock1*^*stl145*^ homozygous mutants injected with *dock1* mRNA robustly express *mbp* in the PLLn (*n* = 13/25). **e)** Quantification of the percent phenotypic classes larvae were scored for *mbp* expression in the PLLn at 3 dpf. Control = pooled uninjected and phenol red injected larvae. **f)** A schematic of the Dock1 protein with the locations of the *stl366*, *stl365*, and *stl145* lesions indicated. **g-j)** Lateral views of *mbp* expression by WISH at 3 dpf. Arrowheads indicate the PLLn. Asterisks indicate the CNS. Inset panels show a magnified view of PLLn. Scale bars = 100 μm. **g)** *dock1*^*stl365*^ homozygous mutants (*n* = 20) and **h)** *dock1*^*stl366*^ homozygous mutants exhibit reduced *mbp* expression in the PLLn (*n* = 15/16). **i)** *dock1*^*stl145/stl365*^ compound heterozygotes and **j)** *dock1*^*stl145/stl366*^ compound heterozygotes exhibit reduced *mbp* expression in the PLLn. **k, l)** Quantification of WISH for *mbp* from *dock1*^*stl365*^ **(k)** and *dock1*^*stl366*^ **(l)** in-crosses based on phenotypic classes and genotypes for the respective lesions. * *p* < 0.05, *** *p* < 0.001, **** p < 0.0001, Chi-squared analysis

An *N*-ethyl-*N*-nitrosourea-based forward genetic screen to identify novel genetic regulators of myelination [29, 30] uncovered a mutant, allele designation *stl145,* that exhibits reduced *mbp* expression in the PNS by whole mount in situ hybridization (WISH). Reduction of *mbp* expression, scored qualitatively in the posterior lateral line nerve (PLLn) (Additional file 1: Figure S1), is most striking at 3 days post fertilization (dpf) (Fig. 1a, b), during early stages of PNS myelination. At 5 dpf, *mbp* expression has increased in the *stl145* mutant PLLn, but is still reduced compared to sibling controls (Fig. 1c, d). To identify the causative mutation, we employed whole genome sequencing (WGS) of DNA pools from phenotypically mutant and phenotypically wild-type siblings. Analysis of WGS data [30] showed the causative mutation was located on chromosome 12 (Fig. 1e). Within the most highly linked region of chromosome 12, the most likely causative mutation was a C-to-T transition resulting in a premature stop codon within the Rac1 binding domain (DHR2 domain) encoding region of *dock1* (Fig. 1f, g). Genotyping revealed that *dock1*^*stl145/stl145*^ homozygous mutations corresponded to decreased levels of *mbp* expression in the PNS at 3 dpf (*p* < 0.0001) and at 5 dpf (p < 0.0001) (Fig. 1h-j). To definitively demonstrate that this premature stop codon was causative for the *stl145* phenotype, we performed a rescue experiment with wild-type *dock1* synthetic mRNA. Injection of 120 pg of full-length synthetic *dock1* mRNA into 1-cell stage embryos derived from an intercross of *dock1*^*stl145*^ heterozygotes suppressed the *mbp* phenotype in *stl145* mutants at 3 dpf (*p* = 0.0001) (Fig. 2 a-e). Additionally, we generated two new alleles of *dock1* using TALENs. The *stl365* allele generates a premature stop codon in the DHR2 domain, similar to the *stl145* allele (Fig. 2 f; Additional file 2: Figure S2 A,B). The *stl366* allele causes a premature stop codon generated just after the SH3 domain (Fig. 2 f; Additional file 2: Figure S2 C,D). Both alleles exhibit decreased *mbp* expression in the PNS (*p* < 0.0001) and fail to complement with the *stl145* allele (*dock1*^*stl145/stl365*^ = 4/4, *dock1*^*stl145/stl365*^ = 6/6; Fig. 2 g-l). To confirm that *dock1* is expressed in Schwann cells of zebrafish in addition to mammalian Schwann cells [20], RT-PCR for *dock1* was performed on cDNA from adult PLLn, which is enriched in Schwann cell nuclei. This analysis showed that *dock1* is expressed in the PLLn (Additional file 2: Figure S2 E,F). Together, these results confirm that the *stl145* phenotype is the result of the premature stop codon in *dock1*. The phenotype of these new mutants suggests a previously unappreciated role of Dock1 in Schwann cell development.

### Schwann cell myelination is significantly reduced in *dock1*^*stl145*^ mutants at early stages

**Fig. 3:**
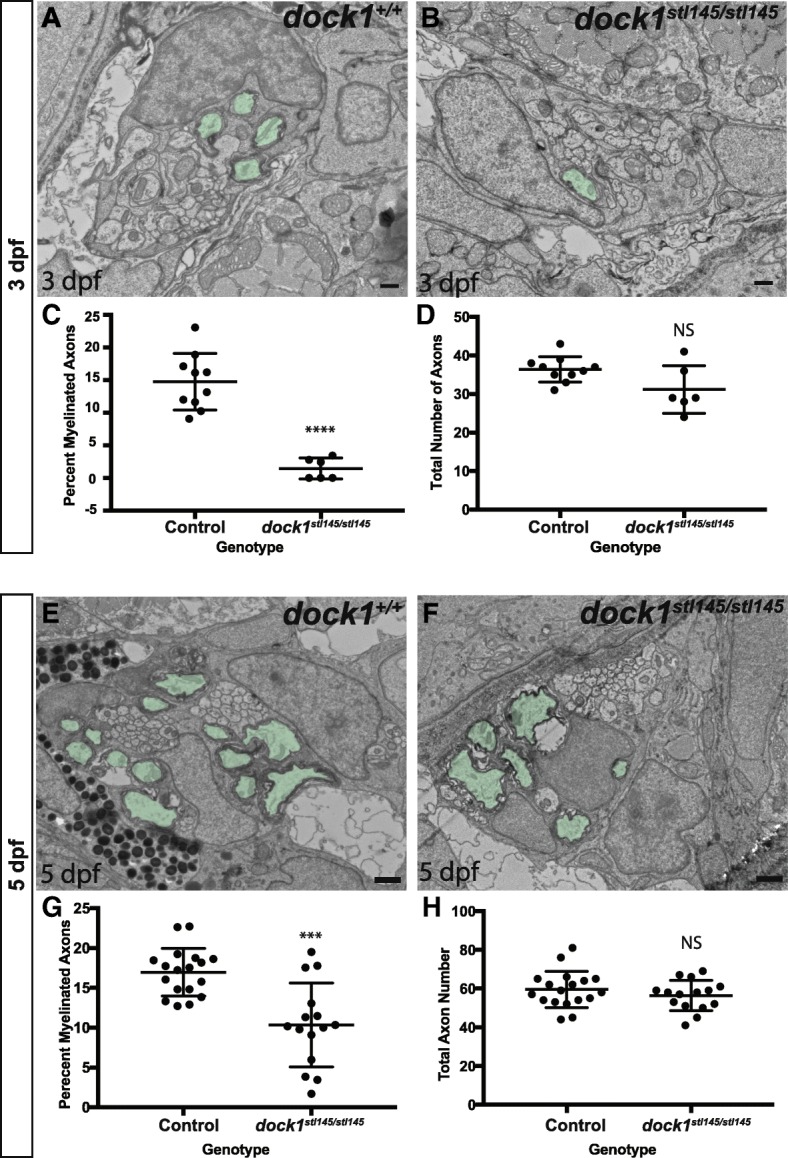
PNS myelination is significantly reduced in *stl145* mutants. **a, b)** TEM of a cross-section of the PLLn at 3 dpf. Myelinated axons are pseudocolored in green. Scale bars = 500 nm. **a)** Axons in wild-type PLLn begin to be myelinated while **b)** *dock1*^*stl145*^ homozygous mutant PLLn exhibits fewer myelination of axons. **c)** Quantification of the percent myelinated axons shows a significant difference between control (*n* = 6 animals, 10 nerves) and *dock1*^*stl145*^ mutants (*n* = 4 animals, 6 nerves). **d)** Quantification of the total number of axons (NS, *p* = 0.0983). **e, f)** Quantification of a cross-section of the PLLn at 5 dpf. Myelinated axons are pseudocolored in green. Scale bars **=** 500 nm. **e)** The PLLn of a wild-type larva contains numerous myelinated axons whereas **f)** a *dock1*^*stl145*^ homozygous mutant PLLn contains fewer myelinated axons. **g)** Quantification of the percent myelinated axons shows a significant difference between control (*n* = 11 animals, 18 nerves) and *dock1*^*stl145*^ mutants (*n* = 9 animals, 15 nerves). **h)** Quantification of the total number of axons (NS, *p* = 0.3031). Bars represent means ± SD. ***p < 0.001, ****p < 0.0001, unpaired *t* Test with Welch’s correction

We next investigated which stages of Schwann cell development are affected in *dock1*^*stl145*^ mutants. To interrogate if the decrease of *mbp* expression in *dock1*^*stl145*^ mutants is the result of decreased myelination, we employed TEM to analyze the ultrastructure of the PLLn at 3 dpf and 5 dpf. At 3 dpf, consistent with WISH for *mbp*, the percentage of myelinated axons in the PLLn of *dock1*^*stl145*^ mutants is significantly reduced compared to siblings (*p* < 0.0001), while the number of axons is not significantly altered (*p* = 0.0983) (Fig. 3a-d). At 5 dpf, *dock1*^*stl145/stl145*^ mutant axon number is similarly unaffected (*p* = 0.3031) while mutants did exhibit a significant decrease in the percentage of myelinated axons compared to controls (*p* = 0.0003), although this phenotype is more variable compared to the mutant phenotype at 3 dpf (Fig. 3 e-h). *dock1*^*stl145/+*^ larvae do not exhibit decreased myelination compared to wild-type siblings at 3 dpf (*p* = 0.6549 percent myelinated axons; *p* = 0.7258 total axon number) or 5 dpf (*p* = 0.7297 percent myelinated axons; *p* = 0.6924 total axon number); thus, wild-type and heterozygous siblings were combined as controls. The presence of myelinated axons at 5 dpf in *dock1*^*stl145/stl145*^ larvae, although fewer in number compared to controls, illustrates that Schwann cells do possess the capability to myelinate axons in *dock1* mutants. Maternal zygotic (MZ) *dock1*^*stl145*^ mutants at 5 dpf also exhibit a reduction in the number of myelinated axons compared to MZ *dock1*^*stl145*^ heterozygotes (*p* = 0.0183) (Additional file 3: Fig. S3 a-d). No significant difference in the percent myelinated axons was observed between zygotic *dock1*^*stl145*^ mutants and MZ *dock1*^*stl145*^ mutants (*p* = 0.8300). To test if a defect in myelination is consistent between alleles, we performed TEM on *dock1*^*stl366*^ mutants and siblings. These mutants also display a significantly decreased percentage of myelinated axons at 3 dpf (*p* = 0.0072) with no significant difference in axon number (*p* = 0.3775) (Additional file 3: Figure S3 E-H). A slight reduction in the percent myelinated axons of the PLLn persists at 21 dpf in MZ *dock1*^*stl145*^ mutants (*p* = 0.0155), while axon number (*p* = 0.5831) and Schwann cell nuclei number (*p* = 0.1583) are not significantly altered compared to MZ *dock1*^*stl145*^ heterozygous controls (Additional file 4: Figure S4). These results show that *dock1* mutations lead to reductions in the number of myelinated axons, and that these effects are more pronounced at early stages of development.

### Neither Schwann cell migration nor number are affected in *dock1*^*stl145*^ mutants

**Fig. 4:**
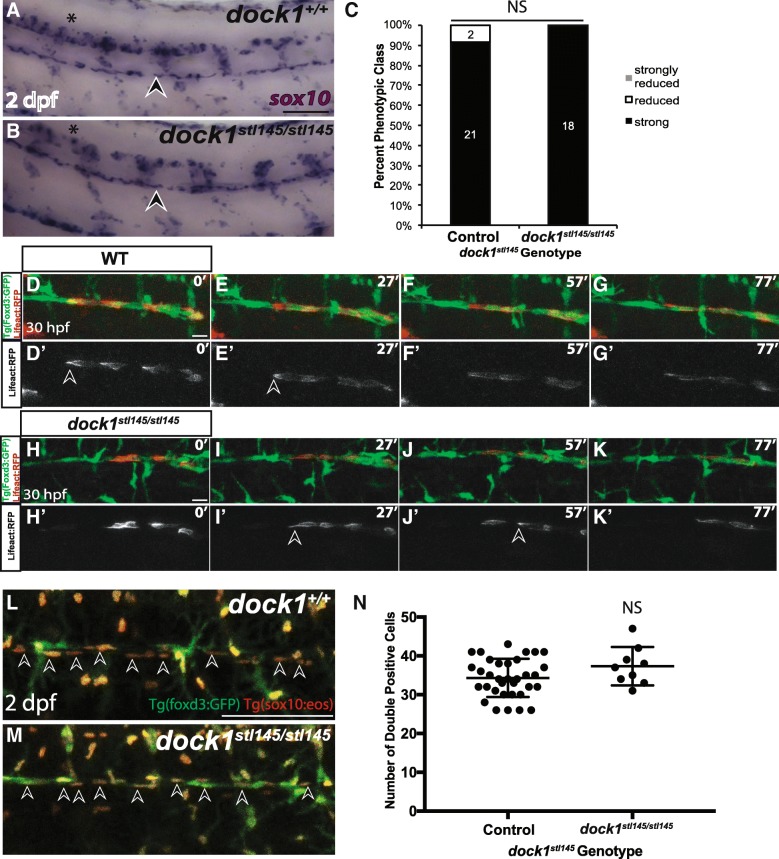
Schwann cell migration and number is not affected in *stl145* mutants. **a, b)** Lateral view of WISH for *sox10* at 2 dpf. Arrowheads indicate the PLLn. Asterisks indicate the CNS. **a)** Strongly expressing *sox10* positive cells are located throughout the PLLn in control larvae (*n* = 21/23), similar to **b)** *dock1*^*stl145*^ homozygous mutant larva (*n* = 18). **c)** Quantification of WISH for *sox10* at 2 dpf based on phenotypic classes and genotypes for the *stl145* lesion shows no significant difference in expression (*p* = 0.3522, Bars represent means ± SD; Chi-squared analysis). **d, g’)** Still images from time-lapse imaging from 30 to 31.5 hpf in *Tg(foxd3:gfp)* wild-type larvae injected with *sox10:Lifeact-RFP*. Prime panels show Lifeact-RFP strongly localized at the back of migrating Schwann cells (arrowheads). Scale bars = 20 μm. **h-k′)** Still images from time-lapse imaging from 30 to 31.5 hpf in *Tg(foxd3:gfp) dock1*^*stl145/stl145*^ larvae injected with *sox10:Lifeact-RFP*. Prime panels show Lifeact-RFP strongly localized at the back of migrating Schwann cells (arrowheads). **l, m)** Lateral view of PLLn in 2 dpf larvae containing *Tg(foxd3:gfp)* and *Tg(sox10(4.9):nls-eos)*. Arrows point to examples of double positive Schwann cells. Counting the number of Schwann cells double positive for GFP and RFP in **l)** control (*n* = 34) and **m)** *dock1*^*stl145*^ homozygous mutants (n = 9). Scale bars = 100 μm. **n)** Quantification of the number of Schwann cells within a defined region of the PLLn revealed no significant difference in Schwann cell number (NS, *p* = 0.1360). Bars represent means ± SD; unpaired *t* Test with Welch’s correction

To understand why myelination is decreased in *dock1*^*stl145*^ mutants at 3 dpf, we examined earlier stages of Schwann cell development, beginning with SCPs. Importantly, global development of *dock1*^*stl145*^ mutants is normal and overt PLLn defects are not observed (Additional file 5: Figure S5 A-F). Acetylated tubulin staining shows that axons extend down the trunk of larvae and neuromast number is not significantly altered (*p* = 0.7518), (Additional file 5: Figure S5 G-I). *dock1*^*stl145/+*^ larvae and wild-type siblings do not exhibit a significant difference in neuromast number (*p* = 0.0727) and were thus combined as a control. Additionally, blood vessels (*kdlr:mcherry* positive) and somites (MF 20 positive) can develop in zygotic *dock1*^*stl145*^ mutants (Additional file 5: Figure S5 J-M). Previous studies have demonstrated that Dock1 can regulate cell migration [41], and two other members of the Dock family of GEFs, Dock7 [20] and Dock8 [21] affect Schwann cell migration. Therefore, we examined if cell migration in *dock1*^*stl145*^ mutants is perturbed by performing WISH for *sox10*, which marks all stages of Schwann cell development, including SCPs. At 2 dpf, SCPs have migrated and populated the PLLn in control larvae as evidenced by strong and consistent expression of *sox10* along the entire length of the PLLn (Fig. 4 a). We found that *dock1*^*stl145/stl145*^ mutants also exhibit consistent and strong expression of *sox10* along the PLLn (*p* = 0.3522), demonstrating that SCP migration is not impaired and that Schwann cells populating the PLLn are thus poised to myelinate (Fig. 4b, c). Dock1 has been shown to regulate the actin cytoskeleton in other systems; therefore, we hypothesized that actin cytoskeletal dynamics might be altered during SCP migration in *dock1*^*stl145*^ mutants. We performed live-imaging of the PLLn in *tg(foxd3:gfp); dock1*^*stl145*^ wild-type, heterozygous, or mutant larvae that also mosaically expressed *sox10:Lifeact-RFP*, which binds and fluorescently labels F-actin in cells expressing *sox10* [42]*.* Live-imaging from ~ 30–33 hpf did not reveal any overt defects in migration or in actin cytoskeleton localization (Fig. 4d-k; Additional file 6: Movie S1, Additional file 7: Movie S2, Additional file 8: Movie S3 and Additional file 9: Movie S4). In both control and *dock1*^*stl145*^ mutant larvae, F-actin was consistently localized to the back of migrating SCPs. To our knowledge, this is the first time live actin dynamics have been reported in migrating SCPs in vivo. High-resolution still images also show LifeAct distributed throughout the cell with the highest concentration at the back of SCPs (Additional file 10: Figure S6).

Because migration is not affected in *dock1*^*stl145*^ mutants, we examined whether decreased myelination in *dock1*^*stl145*^ mutant nerves was the result of fewer Schwann cells. To do this, we generated and analyzed 2 dpf double transgenic *tg(foxd3:gfp);tg(sox10:nls-eos);dock1*^*stl145*^ larvae. The *sox10:nls-eos* transgene enabled manual counting of Schwann cell nuclei along the PLLn, while the *foxd3:gfp* transgene provided a co-label to ensure Schwann cell identity. Counting the number of double positive cells at 2 dpf showed that *dock1*^*stl145/+*^ and wild-type siblings do not exhibit a significant difference in cell number (*p* = 0.2218) and were thus combined as the control group. No significant difference in the number of Schwann cells between mutants and control siblings was observed (*p* = 0.1243), suggesting that a reduction in Schwann cell number is not a contributing factor to decreased myelination of the PNS in *dock1*^*stl145*^ mutants (Fig. 4 l-n). Overall, these experiments demonstrate that SCP migration and number are not overtly affected in *dock1*^*stl145*^ mutants.

### Defects in Schwann cell development are first observed during radial sorting and myelination initiation

**Fig. 5:**
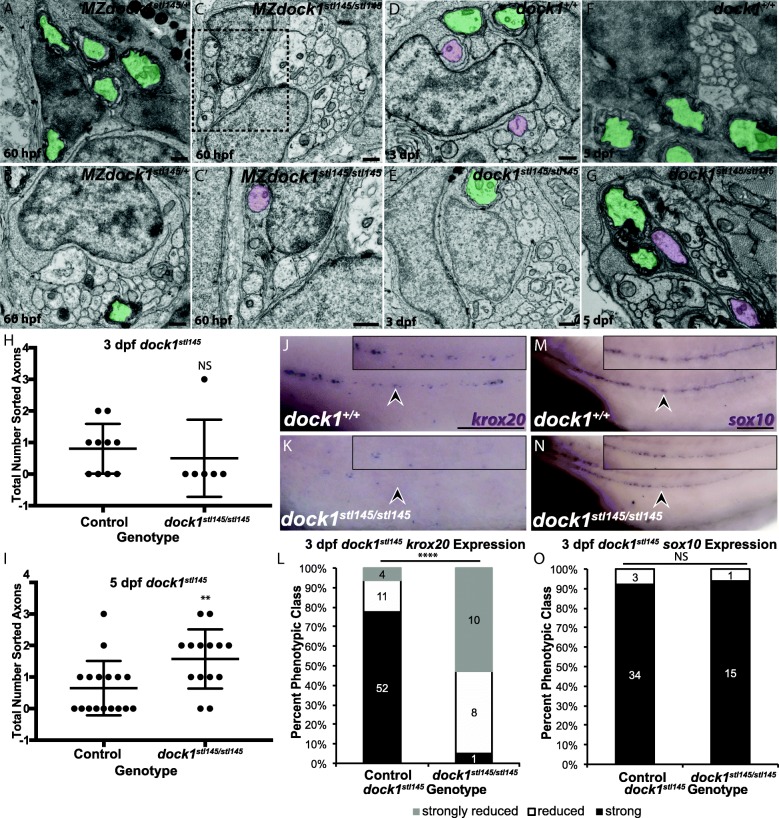
*stl145* mutants exhibit delays in radial sorting and decreased expression of *krox20.* **a-g)** TEM of cross-sections of the PLLn. Myelinated axons are pseudocolored in green and axons associated with promyelinating Schwann cells are pseudocolored in purple. Scale bars = 500 nm. **a, b)** Micrographs from the same PLLn within a MZ*dock1*^*stl145*^ heterozygote show Schwann cells myelinating axons at 60 hpf. **C)** An MZ*dock1*^*stl145*^ homozygous larva does not have myelinated axons at 60 hpf, but Schwann cells are extending processes into axon bundles. **C′)** Magnification of inset from **C** shows an axon surrounded by a pro-myelinating Schwann cell. **d, e)** Schwann cells can myelinate and sort axons at 3 dpf in wild-type (n = 6 animals, 10 nerves) and mutant larvae (*n* = 4 animals, 6 nerves). **f, g)** More sorted axons are present in mutants (*n* = 8 animals, 15 nerves) at 5 dpf compared to controls (*n* = 11 animals, 17 nerves). Quantification of the number of sorted axons at **h)** 3 dpf and **i)** 5 dpf shows a statistical difference at 5 dpf (unpaired *t* Test with Welch’s correction). **j, k)** Lateral view of WISH for *krox20* at 3 dpf. Arrowheads indicate PLLn. Inset panels show a magnified view of the PLLn. Scale bar = 50 μm. **j)** *krox20* is expressed along the PLLn of control larvae (*n* = 67) whereas **k)** *dock1*^*stl145*^ homozygous mutants express little to no *krox20* along the PLLn (n = 18/19). **l)** Quantification of WISH for *krox20* at 3 dpf based on phenotypic classes and genotypes for the *stl145* lesion (*p* < 0.0001, Chi-squared analysis). **m, n)** Lateral view of WISH for *sox10* at 3 dpf. Arrowheads indicate the PLLn. Inset panels show a magnified view of the PLLn. Scale bar = 50 μm. **m)** Control larvae exhibit *sox10* positive Schwann cells along the PLLn (*n* = 37) similar to **N)** *dock1*^*stl145*^ homozygous mutants (*n* = 16). **o)** Quantification of WISH for *sox10* at 3 dpf based on phenotypic classes and genotypes for the *stl145* lesion (*p* = 0.8141, Chi-squared analysis). Bars represent means ± SD, **p < 0.001, **** *p* < 0.0001

Given that the *dock1*^*stl145*^ mutation does not alter SCP migration, we next asked if Schwann cell development was affected at the immature and pro-myelinating Schwann cell stages using TEM. At 60 hpf, Schwann cells in MZ *dock1*^*stl145*^ heterozygous siblings have begun to myelinate axons whereas MZ *dock1*^*stl145*^ mutants are extending processes into axon bundles and can be found in the promyelinating, but not myelinating state (Fig. 5 a-c′). This phenotype suggests that radial sorting by Schwann cells is delayed in in *dock1*^*stl145*^ mutants. We then hypothesized that radial sorting delays at 3 dpf in *dock1*^*stl145*^ mutants might result in higher numbers of Schwann cells associated with axons in a 1:1 ratio at 5 dpf as more Schwann cells have entered the pro-myelinating state. Indeed, compared to 3 dpf, a greater number of Schwann cells are associated in a 1:1 ratio with axons in *dock1*^*stl145*^ mutants (3 dpf: *p* = 0.6068; 5dpf: *p* = 0.0086) (Fig. 5 d-i). To further test if Schwann cells are developmentally delayed at the pro-myelinating state, we examined expression of *krox20* (*egr2*), a transcription factor that initiates expression of myelin associated genes. By WISH, *krox20* expression is significantly decreased along the PLLn of *dock1*^*stl145/stl145*^ mutants compared to wild-type and heterozygous control siblings at 3 dpf (*p* < 0.0001), demonstrating that *dock1*^*stl145*^ mutant Schwann cells are developmentally delayed compared to their siblings (Fig. 5 j-l). This reduction in *krox20* expression is not a result of an absence of Schwann cells because *sox10* positive Schwann cells are present along the PLLn by WISH at 3 dpf (*p* = 0.8141) (Fig. 5 m-o). Together, these data show that *dock1*^*stl145*^ mutants exhibit delays in development that begin during radial sorting and extend throughout initial myelination of the PNS in zebrafish.

## Discussion

A critical component of Schwann cell development is the remodeling of the cytoskeleton to promote shape changes to facilitate proper myelination of the PNS [3, 43]. Although some of the intracellular components involved in cytoskeletal rearrangements have been identified, such as Rac1, the full complement of proteins involved in this process has not been comprehensively defined. Through a forward genetic screen in zebrafish, we identified an early stop codon in the Rac1 binding domain of *dock1*, an atypical GEF, that causes decreased *mbp* expression in the PNS at 3 dpf and 5 dpf. Rescue experiments and complementation tests with two newly engineered alleles of *dock1* confirmed that mutations in *dock1* result in decreased *mbp* expression.

TEM analysis showed fewer myelinated axons in mutants at 3 dpf, 5 dpf, and 21 dpf, whereas axon number is not significantly affected at any stage assessed. However, we did note that several unmyelinated axons in some mutant nerves were abnormally large in diameter and had many mitochondria (data not shown). We demonstrated that reduced *mbp* expression and the reduction of myelinated axons in mutants is not caused by absence or loss of Schwann cell number. While two other members of the Dock1-family of atypical GEFs, Dock7 and Dock8, affect SCP migration in mammals [20, 21], this does not appear to be the case of Dock1 in zebrafish. This is not entirely unexpected, since loss of Rac1 in mouse Schwann cells did not affect Schwann cell migration [5, 6]. Although overt defects in migration were not detected using live-imaging, these experiments enabled visualization of F-actin localization, which showed that F-actin is localized at the back of migrating Schwann cells. This live-imaging data with Lifeact supports previously reported data from 3D culture of Schwann cells showing that migrating Schwann cells in vivo move in an amoeboid-like fashion [44], as contractions seem to occur at the back of the cell. In the future, it will be interesting to generate *dock7* and *dock8* zebrafish genetic mutants and observe how migration and F-actin localization is affected in SCPs.

Although a significant reduction in the percent myelinated axons is observed at 5 dpf, Schwann cells in *dock1*^*stl145*^ mutants do have the capability to myelinate axons, suggesting that *dock1* is involved in the timing of myelination onset. It is also possible that other Dock1 family members compensate for *dock1* loss of function in our mutants. Further experiments are needed to determine if Dock1 functions in a Schwann cell-autonomous or non-cell-autonomous manner. Consistent with data showing that *dock1* mutant Schwann cells are delayed in radial sorting and myelination, expression of *krox20*, a transcription factor essential for expression of myelin genes, is decreased in *dock1*^*stl145*^ mutant nerves. Importantly, Schwann cell number is not affected in *dock1*^*stl145*^ mutants and overall PLLn development is not affected in *dock1*^*stl145*^ mutants compared to controls, as determined by acetylated tubulin staining and counting neuromast number. Combined, these data demonstrate that Schwann cell radial sorting and myelination are delayed in *dock1* mutants.

**Fig. 6:**
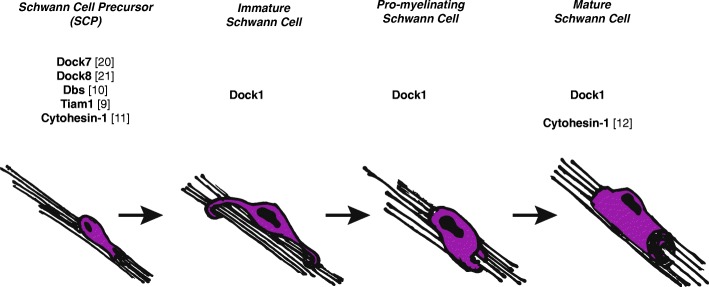
Roles of GEFs in Schwann cell development. Several canonical and atypical GEFs have been characterized in Schwann cell development, primarily during Schwann cell migration. Dock1 functions either cell autonomously or non-cell autonomously to regulate immature to myelinating stages of Schwann cell development

How might Dock1 regulate Schwann cell radial sorting and myelination? GEFs are proposed to aid in regulating the spatial and temporal activation of RhoGTPases, such as Rac1 [8]. During the course of development, Schwann cells undergo unique and critical cell shape changes during migration, radial sorting, and myelination. We hypothesize that to regulate the cytoskeletal rearrangements necessary for such functions, Schwann cells utilize a “tool-kit” of GEFs – both canonical and atypical – to activate RhoGTPases, which subsequently remodel the cytoskeleton. For example, Dock7 and Dock8, in addition to canonical GEFs, Dbl’s big sister (Dbs) [10] and Tiam1 [9], have already been shown to regulate SCP migration. Dock1 may be activated after Schwann cells have migrated to subsequently promote radial sorting and early myelination. Because few GEFs have been shown to play a role in Schwann cell development in vivo, important next steps in elucidating the discrete signals necessary for development will be to define the repertoire of active GEFs, particularly after SCP migration (Fig. 6). RhoGTPases, like Rac1, are ubiquitous and important for initiating cytoskeletal rearrangements as well as other cell biological processes; therefore, different GEFs may be utilized to activate RhoGTPases cell-specifically. Previously, it has been shown that Rac1 activation regulates Schwann cell radial sorting and myelination [6] in addition to promoting the transition from Schwann cell migration to radial sorting [5]. Because Dock1 has been shown to specifically bind and activate Rac1 in various biological contexts, we hypothesize that if Dock1 functions cell-autonomously, it may be one of many GEFs that activate Rac1 in Schwann cells. Multiple GEFs working together in concert could increase the total levels of activated Rac1 during a critical period in Schwann cell development to enable the process extensions necessary for radial sorting and myelination. One explanation of our data is that Schwann cell radial sorting and myelination is slower in *dock1* mutants because activated Rac1 levels have not reached a critical threshold to promote these processes. In the future, as better in vivo Rac1 sensors are developed, it will be interesting to test this hypothesis. Additionally, Dock1 may function redundantly with other GEFs. As radial sorting and myelination are critical for PNS health, many GEFs may converge on the same pathway such that if only one GEF is dysfunctional, radial sorting and myelination can still proceed, albeit at a slower rate. Alternatively, Dock1 may function in neurons and mutations in *dock1* may indirectly affect Schwann cell development.

The cell autonomy of Dock1 function and the upstream signals that trigger Dock1 activation remain to be elucidated. Dock1 has a DHR1 domain that can bind phosphatidylinositols [45] located in the cell membrane, making Dock1 is an attractive candidate to serve as a link between cell surfaces receptor and the cytoskeleton. Additionally, Dock1 could be a representative of a class of drug targets for diseases affecting peripheral myelin, especially because GEFs may contribute to myelination disease states in human patients [4, 46–48]. Although RhoGTPases are critical for cytoskeletal rearrangements, their ubiquity in many cell types limits their ability to serve as useful therapeutic drug targets. Alternatively, GEFs, particularly atypical GEFs like Dock1, could open a door to indirectly affect RhoGTPases in a more cell-specific manner and thus influence the cell shape changes that promote proper Schwann cell development and myelination.

## Conclusions

In this study, we demonstrate that mutations in an atypical GEF, *dock1*, result in defects in Schwann cell radial sorting and myelination. Schwann cells are slower to extend processes into axon bundles and subsequently myelinate fewer axons. Schwann cell number and migration are not affected in these mutants; however, Schwann cells in *dock1* mutants fail to robustly express markers such as *mbp* and *krox20* in early development, suggesting that *dock1* aids in the temporal regulation of Schwann cell radial sorting and development. Moreover, Dock1 may represent a link between extracellular signals and the intracellular cytoskeletal rearrangements necessary for radial sorting and myelination.

## Additional files

Additional file 1: **Figure S1. A-C)** Lateral view of WISH for *mbp*. Arrowheads indicate the PLLn. Asterisks indicate the CNS. **A)** Representative image of a PLLn scored as “strong” expression, with *mbp* strongly and continuously expressed along PLLn **B)** as “reduced” expression, with reduced but consistent *mbp* expression along PLLn and **C)** as “strongly reduced” with patchy *mbp* expression along the PLLn. (PDF 846 kb)

Additional file 2: **Figure S2. A)** The *stl365* allele was generated by a TALEN that resulted in one base pair insertion causing an early stop. **B)** Genotyping assay for the *stl365* lesion. The PCR amplified product is digested with EcoRV and run on a 3% agarose gel. **C)** The *stl366* allele was generated by a TALEN that resulted in a 13 base-pair deletion causing an early stop. **D)** Genotyping assay for the *stl366* lesion. The PCR amplified product is digested with HpyCH4III and run on a 3% agarose gel. **E)** RT-PCR for *dock1* on adult PLLn cDNA shows *dock1* is expressed in the PLLn. **F)** Control reaction performed with Milli-q water as a substrate. (PDF 362 kb)

Additional file 3: **Figure S3. A-B)** TEM of a cross-section of the PLLn at 5 dpf in MZ siblings. Myelinated axons are pseudocolored in green. Scale bars = 500 nm**. A)** Axons in *MZdock1*^*stl145*^ heterozygotes (*n* = 4 animals, 6 nerves) contain many myelinated axons whereas **B)** *MZdock1*^*stl145*^ mutants have fewer myelinated axons (*n* = 5 animals, 8 nerves). **C)** Quantification of the percent myelinated axons. **D)** Quantification of the total number of axons (NS, *p* = 0.2926). **E-F)** TEM of a cross-section of the PLLn at 3 dpf in *dock1*^*stl366*^ siblings. Myelinated axons are pseudocolored in green. Scale bars = 500 nm. **E)** Schwann cells in control siblings have myelinated more axons (n = 4 animals, 6 nerves) compared to **F)** *dock1*^*stl366*^ homozygous mutant nerves (*n* = 3 animals, 5 nerves). **G)** Quantification of the percent myelinated axons. **H)** Quantification of the total number of axons (NS, *p* = 0.3775). Bars represent means ± SD. *p < 0.05, **p < 0.01, unpaired *t* Test with Welch’s correction. (PDF 1677 kb)

Additional file 4: **Figure S4. A-B)** TEM of a cross-section of the PLLn at 21 dpf in MZ siblings. Scale bars = 10 μm**. (A’-B′)** Magnified images. Scale bars = 2 μm. **A-A’)** Axons in *MZdock1*^*stl145*^ heterozygotes (n = 4 animals, 5 nerves) contain many myelinated axons and **B-B′)** *MZdock1*^*stl145*^ mutants have fewer myelinated axons (*n* = 2 animals, 3 nerves). **C)** Quantification of the percent myelinated axons. **D)** Quantification of the total number of axons (NS, *p* = 0.5831). **E)** Quantification of the total number of Schwann cell nuclei (NS, *p* = 0.1583). Bars represent means ± SD. *p < 0.05, unpaired *t* Test with Welch’s correction. (PDF 2275 kb)

Additional file 5: **Figure 5.** Gross development is normal at 3 dpf comparing **A)** wild-type, **B)** heterozygous, and **C)** mutant larvae from a *dock1*^*stl145*^ intercross. Scale bars = 500 μm**. D-F)** Gross development is normal and swim bladders have inflated at 5 dpf comparing **D)** wild-type, **E)** heterozygous, and **F)** mutant from a *dock1*^*stl145*^ intercross. Scale bars = 500 μm. **G)** Acetylated tubulin shows axons are present and well-fasiculated in both wild-type (n = 3) and **H)** *dock1*^*stl145/stl145*^ mutant larvae (*n* = 9) at 4 dpf. **I)** Neuromast number, detected by DASPEI labeling, did not vary between controls (*n* = 46) or mutants (*n* = 16) at 3 dpf (NS, *p* = 0.7518), indicating that global PLLn development is not affected. Bars represent means ± SD; unpaired *t* Test with Welch’s correction. **J)** *Tg(kdlr:mcherry)* labeling blood vessels at 4 dpf in wild-type and **K)** *dock1*^*stl14/stl145*^ mutants. **L)** MF 20 staining shows defined somite development in wild-type and **M)** *dock1*^*stl14/stl145*^ mutant larvae at 1 dpf. Scale bars = 100 μm. (PDF 5492 kb)

Additional file 6: **Movie S1.** Live-imaging of a *Tg(foxd3:gfp)* wild-type larva (~ 30–33 hpf) injected with *sox10:Lifeact-RFP.* The larva was imaged every 3 min for 3 h. (AVI 8176 kb)

Additional file 7: **Movie S2.** Grayscale single channel movie of Lifeact as seen in Additional file 6: Movie S1. (AVI 6489 kb)

Additional file 8: **Movie S3.** Live-imaging of a *Tg(foxd3:gfp) dock1*^*stl145/stl145*^ larva (~ 30–33 hpf) injected with *sox10:Lifeact-RFP.* The larva was imaged every 3 min for 3 h. (AVI 5595 kb)

Additional file 9: **Movie S4.** Grayscale single channel movie of Lifeact as seen in Additional file 8: Movie S3. (AVI 2810 kb)

Additional file 10: **Figure S6. A)** Zeiss Airyscan image of *Tg(foxd3:gfp)* wild-type and **B)** *Tg(foxd3:gfp) dock1*^*stl145/+*^ larva (~ 30 hpf) injected with *sox10:Lifeact-RFP.* **A’-B′)** Lifeact-RFP localization within Schwann cell precursors. Scale bars = 10 μm. (PDF 557 kb)

## Abbreviations

bp: Base pair
CNS: Central nervous system
dpf: Days post fertilization
GDP: Guanosine diphosphate
GEF: Guanine nucleotide exchange factor
GTP: Guanosine triphosphate
hpf: Hours post fertilization
*mbp*: *Myelin basic protein*
MF 20: Myosin heavy chain antibody
mm: Millimeter
mM: Millimolar
MZ: Maternal zygotic
ng: Nanogram
nl: Nanoliter
PBS: Phosphate buffered saline
PCR: Polymerase chain reaction
pg: Picogram
PLLn: Posterior lateral line nerve
PNS: Peripheral nervous system
PtdIns(3,4,5)P_3_: Phosphatidylinositol (3,4,5)-trisphosphate
SCP: Schwann cell precursor
SD: Standard deviation
TEM: Transmission electron microscopy
WISH: Whole mount in situ hybridization
WT: Wild-type
μg: Microgram
μm: Micrometer
