# Supplementary figures and images for: Mutations in dock1 disrupt early Schwann cell development
Source: Neural Dev. 2018 Aug 8;13:17. doi: 10.1186/s13064-018-0114-9 (PMC6083577; doi:10.1186/s13064-018-0114-9)

**A**

**STRONG**

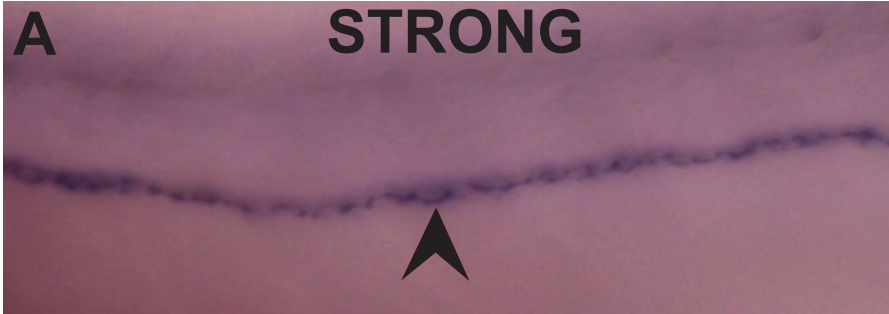

**B**

**REDUCED**

**\***

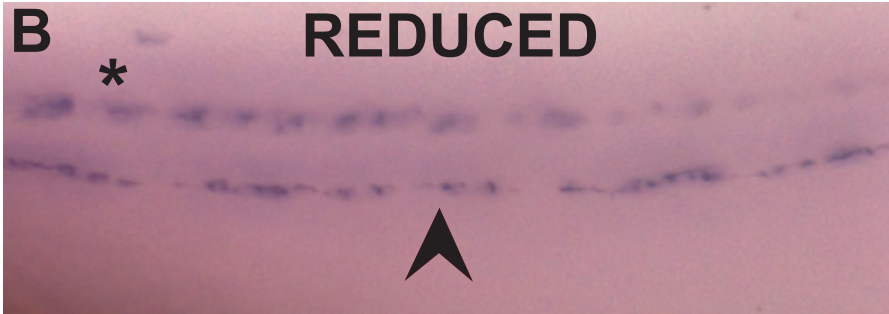

**C \* STRONGLY REDUCED**

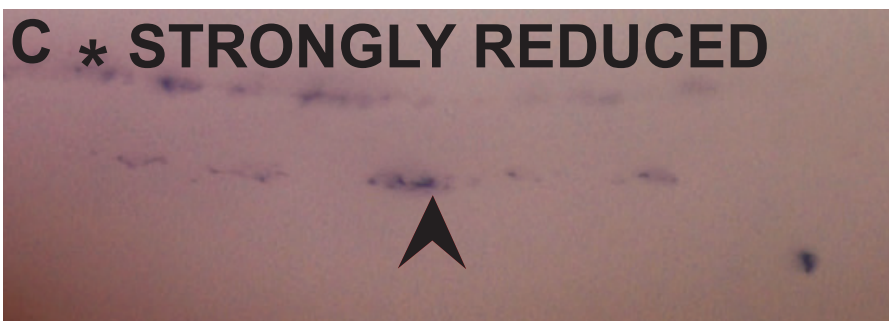

Supplement: Supplementary file 1 — Figure S1. A-C) Lateral view of WISH for mbp. Arrowheads indicate the PLLn. Asterisks indicate the CNS. A) Representative image of a PLLn scored as “strong” expression, with mbp strongly and continuously expressed along PLLn B) as “reduced” expression, with reduced but consistent mbp expression along PLLn and C) as “strongly reduced” with patchy mbp expression along the PLLn. (PDF 846 kb) [file 13064_2018_114_MOESM1_ESM.pdf]

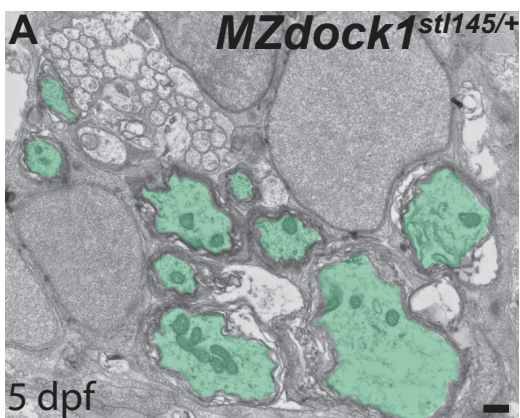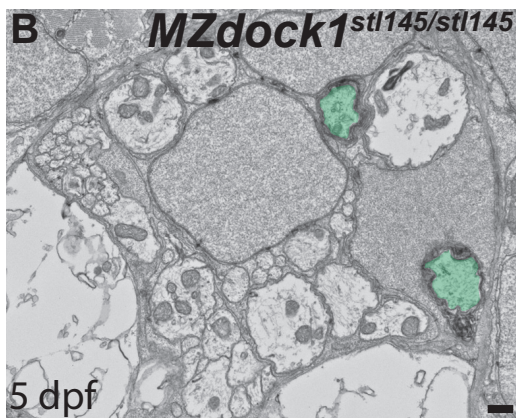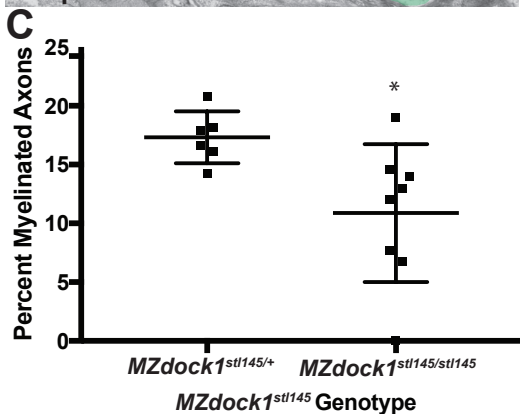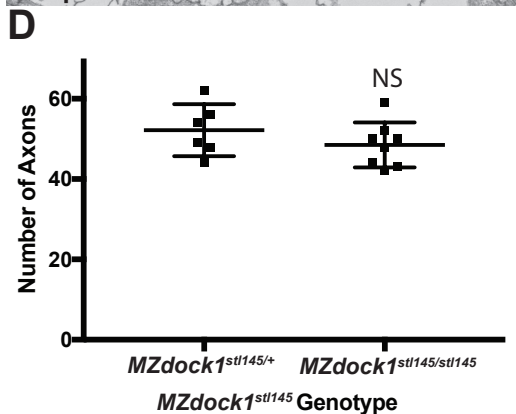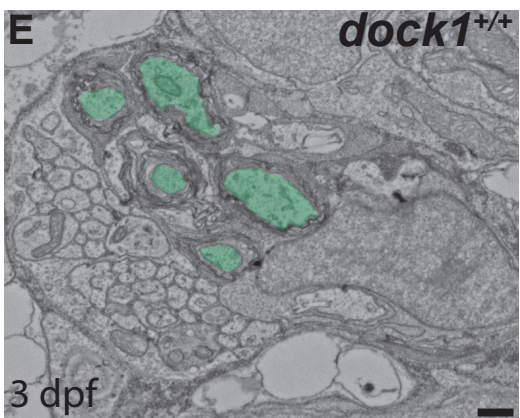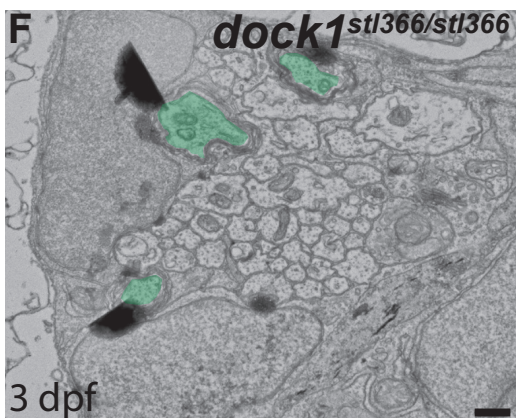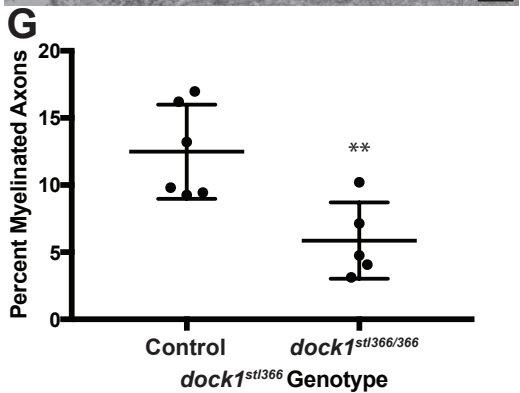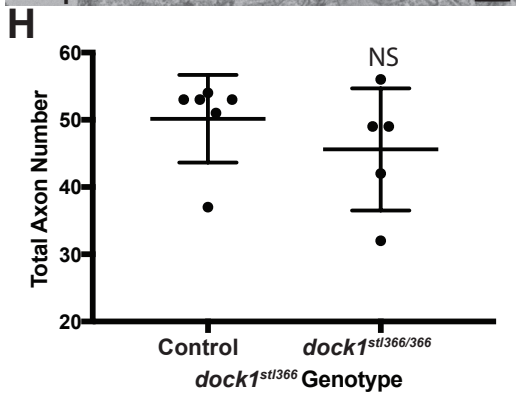

Supplement: Supplementary file 3 — Figure S3. A-B) TEM of a cross-section of the PLLn at 5 dpf in MZ siblings. Myelinated axons are pseudocolored in green. Scale bars = 500 nm. A) Axons in MZdock1stl145 heterozygotes (n = 4 animals, 6 nerves) contain many myelinated axons whereas B) MZdock1stl145 mutants have fewer myelinated axons (n = 5 animals, 8 nerves). C) Quantification of the percent myelinated axons. D) Quantification of the total number of axons (NS, p = 0.2926). E-F) TEM of a cross-section of the PLLn at 3 dpf in dock1stl366 siblings. Myelinated axons are pseudocolored in green. Scale bars = 500 nm. E) Schwann cells in control siblings have myelinated more axons (n = 4 animals, 6 nerves) compared to F) dock1stl366 homozygous mutant nerves (n = 3 animals, 5 nerves). G) Quantification of the percent myelinated axons. H) Quantification of the total number of axons (NS, p = 0.3775). Bars represent means ± SD. *p < 0.05, **p < 0.01, unpaired t Test with Welch’s correction. (PDF 1677 kb) [file 13064_2018_114_MOESM3_ESM.pdf]

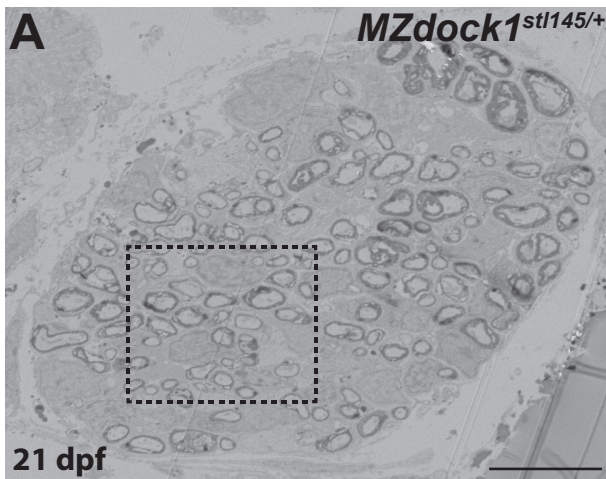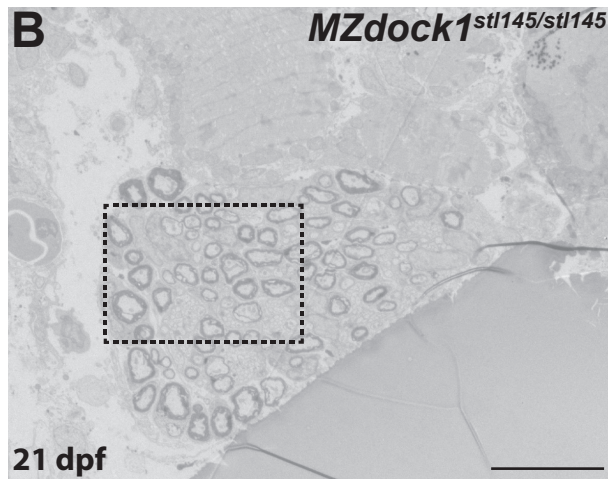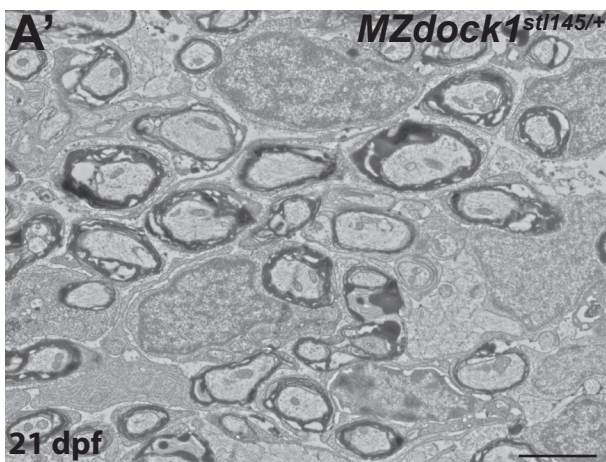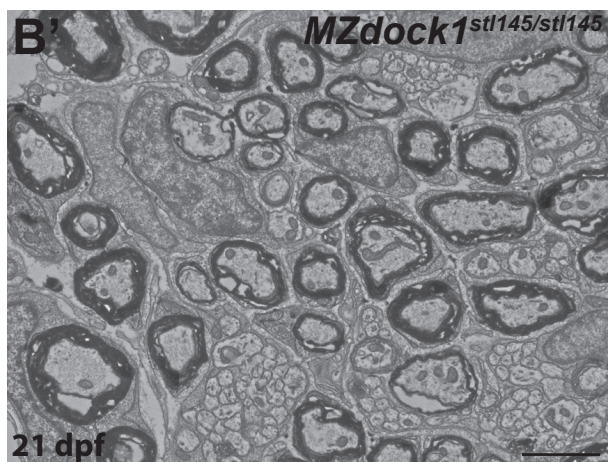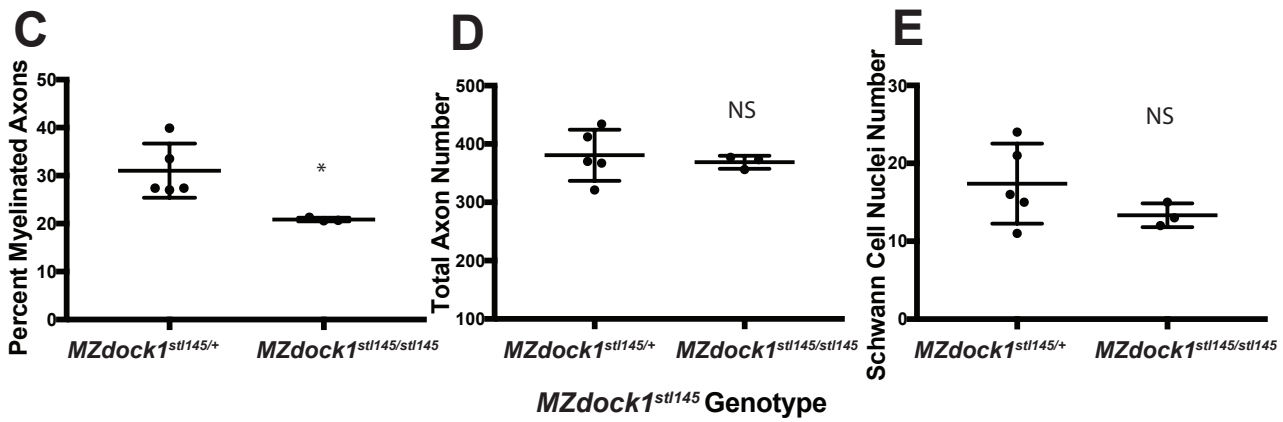

Supplement: Supplementary file 4 — Figure S4. A-B) TEM of a cross-section of the PLLn at 21 dpf in MZ siblings. Scale bars = 10 μm. (A’-B′) Magnified images. Scale bars = 2 μm. A-A’) Axons in MZdock1stl145 heterozygotes (n = 4 animals, 5 nerves) contain many myelinated axons and B-B′) MZdock1stl145 mutants have fewer myelinated axons (n = 2 animals, 3 nerves). C) Quantification of the percent myelinated axons. D) Quantification of the total number of axons (NS, p = 0.5831). E) Quantification of the total number of Schwann cell nuclei (NS, p = 0.1583). Bars represent means ± SD. *p < 0.05, unpaired t Test with Welch’s correction. (PDF 2275 kb) [file 13064_2018_114_MOESM4_ESM.pdf]

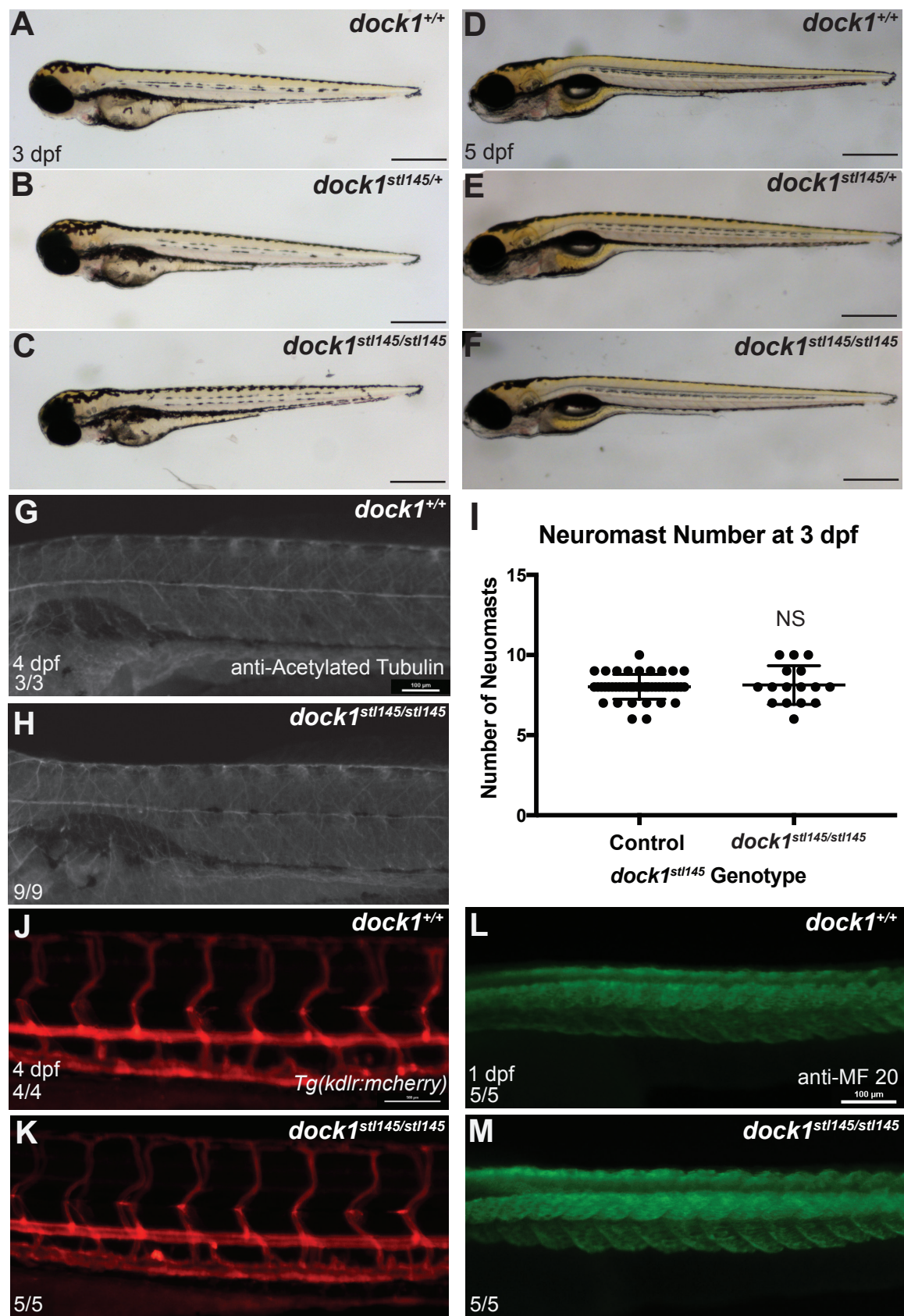

Supplement: Supplementary file 5 — Figure 5. Gross development is normal at 3 dpf comparing A) wild-type, B) heterozygous, and C) mutant larvae from a dock1stl145 intercross. Scale bars = 500 μm. D-F) Gross development is normal and swim bladders have inflated at 5 dpf comparing D) wild-type, E) heterozygous, and F) mutant from a dock1stl145 intercross. Scale bars = 500 μm. G) Acetylated tubulin shows axons are present and well-fasiculated in both wild-type (n = 3) and H) dock1stl145/stl145 mutant larvae (n = 9) at 4 dpf. I) Neuromast number, detected by DASPEI labeling, did not vary between controls (n = 46) or mutants (n = 16) at 3 dpf (NS, p = 0.7518), indicating that global PLLn development is not affected. Bars represent means ± SD; unpaired t Test with Welch’s correction. J) Tg(kdlr:mcherry) labeling blood vessels at 4 dpf in wild-type and K) dock1stl14/stl145 mutants. L) MF 20 staining shows defined somite development in wild-type and M) dock1stl14/stl145 mutant larvae at 1 dpf. Scale bars = 100 μm. (PDF 5492 kb) [file 13064_2018_114_MOESM5_ESM.pdf]

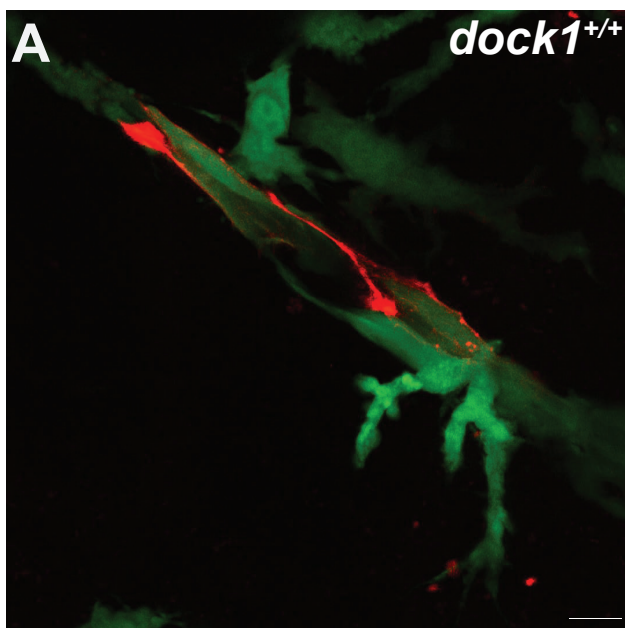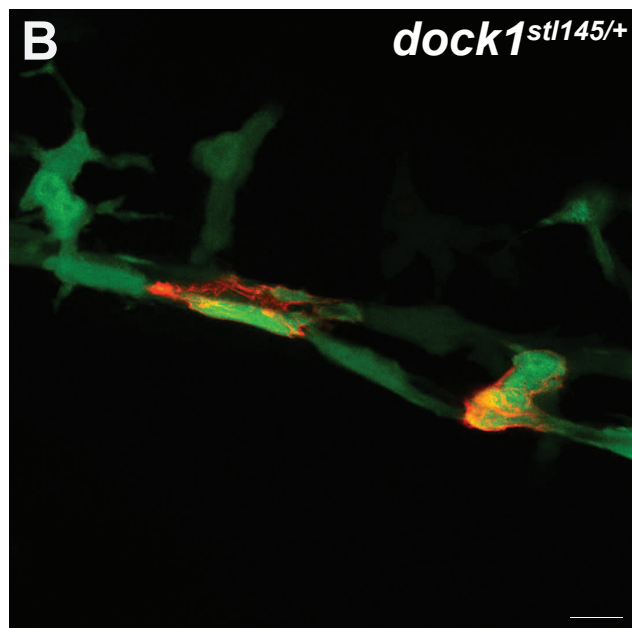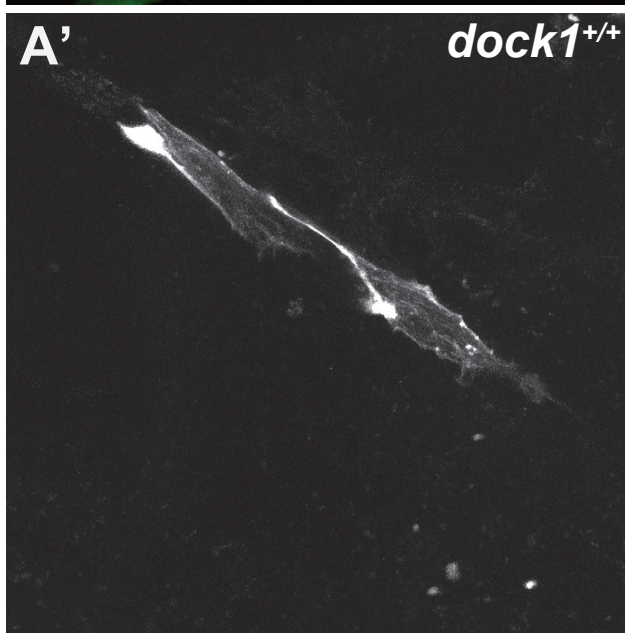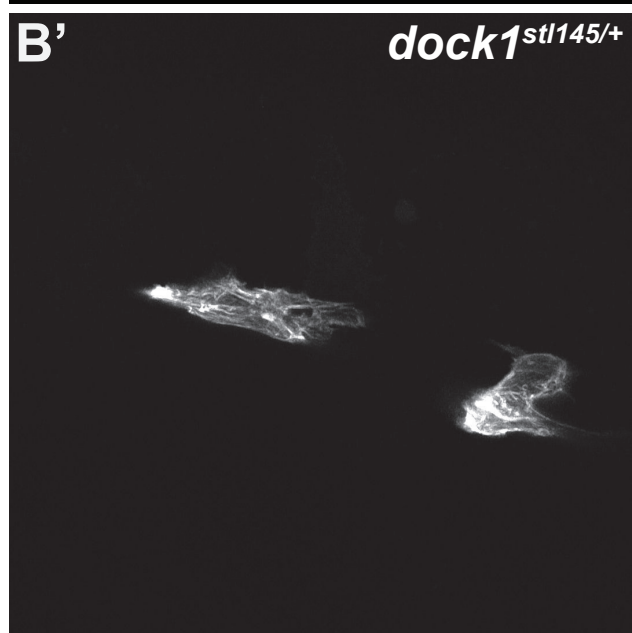

Supplement: Supplementary file 10 — Figure S6. A) Zeiss Airyscan image of Tg(foxd3:gfp) wild-type and B) Tg(foxd3:gfp) dock1stl145/+ larva (~ 30 hpf) injected with sox10:Lifeact-RFP. A’-B′) Lifeact-RFP localization within Schwann cell precursors. Scale bars = 10 μm. (PDF 557 kb) [file 13064_2018_114_MOESM10_ESM.pdf]
