# Supplementary material for: Mutations in dock1 disrupt early Schwann cell development
Source: Neural Dev. 2018 Aug 8;13:17. doi: 10.1186/s13064-018-0114-9 (PMC6083577; doi:10.1186/s13064-018-0114-9)
Supplement: Supplementary file 2 — Figure S2. A) The stl365 allele was generated by a TALEN that resulted in one base pair insertion causing an early stop. B) Genotyping assay for the stl365 lesion. The PCR amplified product is digested with EcoRV and run on a 3% agarose gel. C) The stl366 allele was generated by a TALEN that resulted in a 13 base-pair deletion causing an early stop. D) Genotyping assay for the stl366 lesion. The PCR amplified product is digested with HpyCH4III and run on a 3% agarose gel. E) RT-PCR for dock1 on adult PLLn cDNA shows dock1 is expressed in the PLLn. F) Control reaction performed with Milli-q water as a substrate. (PDF 362 kb) [file 13064_2018_114_MOESM2_ESM.pdf]

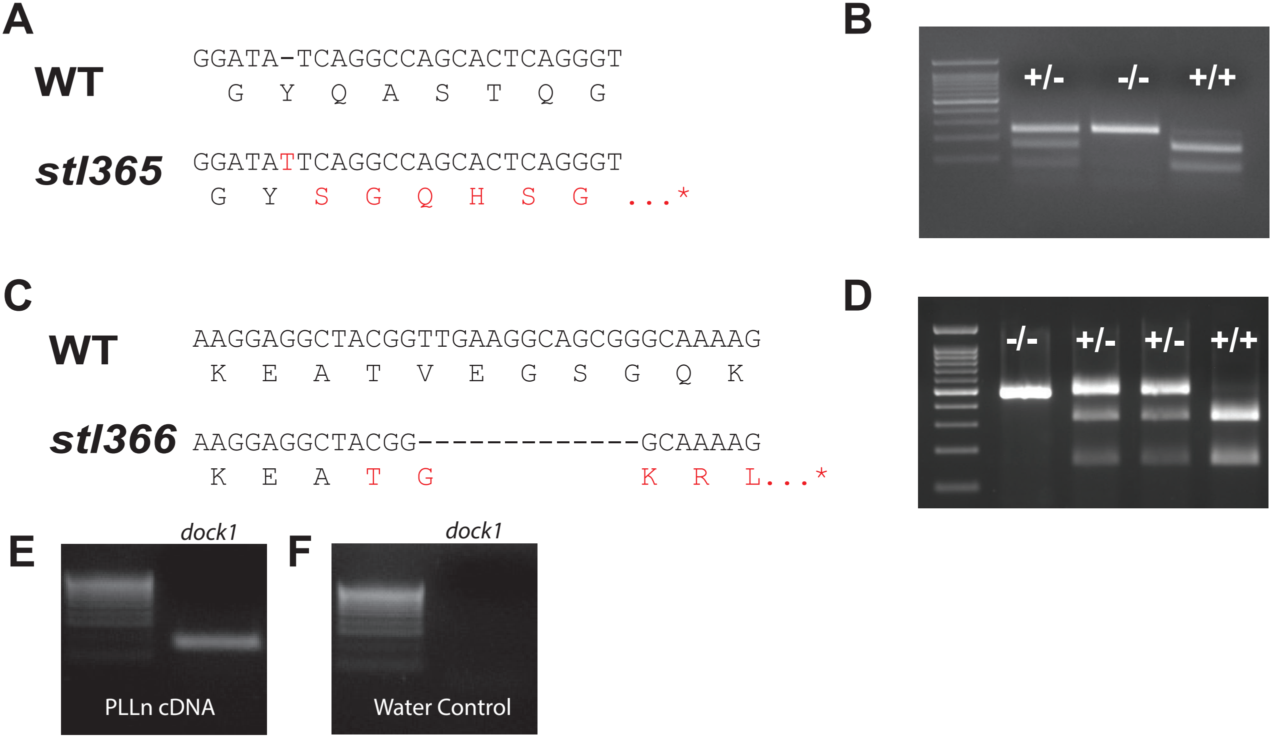

A
WT
GGATA-TCAGGCCAGCACTCAGGGT
G Y Q A S T Q G
stl365
GGATATTCAGGCCAGCACTCAGGGT
G Y S G Q H S G ...*
B
+/-
-/-
+/+
C
WT
AAGGAGGCTACGGTTGAAGGCAGCGGCAAAAG
K E A T V E G S G Q K
stl366
AAGGAGGCTACGG--------------GCAAAAG
K E A T G K R L...*
D
-/-
+/-
+/-
+/+
E
dock1
PLLn cDNA
F
dock1
Water Control
